# Differential proteomic profile of spermatogenic and Sertoli cells from peri-pubertal testes of three different bovine breeds

**DOI:** 10.3389/fcell.2014.00024

**Published:** 2014-06-04

**Authors:** Utkarsh K. Tripathi, Muhammad K. M. Aslam, Shashank Pandey, Samiksha Nayak, Shivani Chhillar, A. Srinivasan, T. K. Mohanty, Prashant H. Kadam, M. S. Chauhan, Savita Yadav, Arumugam Kumaresan

**Affiliations:** ^1^Theriogenology Lab, Livestock Production Management, National Dairy Research InstituteKarnal, India; ^2^Department of Biophysics, All India Institute of Medical SciencesNew Delhi, India; ^3^Embryo Biotechnology Lab, Animal Biotechnology Centre, National Dairy Research InstituteKarnal, India

**Keywords:** bulls, spermatogenic cells, Sertoli cells, proteomics, crossbred, purebred

## Abstract

Sub-fertility is one of the most common problems observed in crossbred males, but the etiology remains unknown in most of the cases. Although proteomic differences in the spermatozoa and seminal plasma between breeds have been investigated, the possible differences at the sperm precursor cells and supporting/nourishing cells have not been studied. The present study reports the differential proteomic profile of spermatogenic and Sertoli cells in crossbred and purebred bulls. Testis was removed by unilateral castration of 12 peri-pubertal bulls (10 months age), four each from crossbred (Holstein Friesian × Tharparkar), exotic purebred [Holstein Friesian (HF)] and indigenous purebred [Tharparkar (TP)] bulls. Spermatogenic and Sertoli cells were isolated and subjected to proteomic analysis. Protein extracts from the Sertoli and spermatogenic cells of each breed were analyzed with 2-dimensional difference gel electrophoresis (2D-DIGE) and analyzed with Decyder™ software. Compared to HF, 26 protein spots were over expressed and 14 protein spots were under expressed in spermatogenic cells of crossbred bulls. Similarly, 7 protein spots were over expressed and 15 protein spots were under expressed in the spermatogenic cells of TP bulls compared to that of crossbred bulls. Out of 12 selected protein spots identified through mass spectrometry, Phosphatidyl ethanolamine binding protein was found to be over expressed in the spermatogenic cells of crossbred bulls compared to TP bulls. The protein, gamma actin was found to be over expressed in the Sertoli cells of HF bulls, whereas Speedy Protein-A was found to be over expressed in Sertoli cells of crossbred bulls. It may be concluded that certain proteomic level differences exist in sperm precursor cells and nourishing cells between breeds, which might be associated with differences in the fertility among these breeds.

## Introduction

Sub-fertility and poor semen quality are the important reasons of dairy bull wastage and the magnitude of these problems are high in crossbred bulls (Vijetha et al., 2014). The males produced through crossing of *Bos taurus* with *Bos indicus* suffer from serious infertility/sub-fertility problems (Mukhopadhyay et al., 2010). The seminal parameters and fertility of the crossbred bulls have been reported to be poor than the indigenous breeds (Khatun et al., 2013). The reason for production of low quality semen in crossbred bulls, even during the best breeding season, has yet to be identified. It is well known that production of fertile sperm is a consequence of normal mitosis and meiosis of germ cell and proper function of germ and Sertoli cells (Johnson, 1991). Sub-fertility and poor semen quality may be attributed to impaired spermatogenesis, endocrine disturbances and alterations in micro-environment of seminiferous tubules (Robaire and Viger, 1995; Hinton and Palladino, 1996). Till date, the subfertility/infertility among the bulls can be recognized at a very later stage only, i.e., after semen collection, freezing and *in vivo* fertility trials, which lead to huge financial loss. Recent developments in analytical techniques (such as proteomics) helped researchers to find out the differences in spermatozoa/seminal plasma of bulls with high and low fertility. Using proteomics, fertility associated markers have been identified in bulls (D'Amours et al., 2010; Park et al., 2012; Soggiu et al., 2013); however these markers can be used only when the bull starts ejaculation of semen. If fertility related markers are identified in testicular cells that are present at younger age, then the bull calves can be screened for the marker at an early age so that the cost involved in bull rearing is reduced. Studying the basic differences in testicular components and their functions between breeds with high and low incidence of infertility would help us to develop tools for early prediction of fertility.

Sertoli cells are the somatic cells of the testes that are essential for spermatogenesis. These cells facilitate the progression of germ cells to spermatozoa through direct contact and by controlling the environment *milieu* within the seminiferous tubules. Recently, we observed that the proportion of Sertoli cells in relation to spermatogenic cells was low in crossbred bulls compared to indigenous bulls (unpublished data). Although quantitative differences in Sertoli and spermatogenic cells between breeds were estimated, possible qualitative differences have not been studied. Proteomic strategies are used to directly investigate reproductive physiopathology, with the aim of providing new tools for the detection of sperm alterations at the protein level (Calvel et al., 2010). Thus, studying the proteomics of Sertoli and spermatogenic cells of crossbred bulls and purebred bulls would improve our understanding on the qualitative differences between these breeds. Previously, few scientists have reported the testicular cell proteomics in humans (Guo et al., 2010), mice (Li et al., 2013), rats (Oh et al., 2014), rhesus monkeys (Wang et al., 2014), pigs (Huang et al., 2005), and fishes (Martyniuk and Alvarez, 2013). But all these studies analyzed the proteomic profile of whole testicular tissue, and not of separate cellular components. Moreover no reports are available about the proteomic profile of various testicular cells in bulls.

We hypothesized that studying the differential expression of certain proteins in spermatogenic and Sertoli cells of different breeds may provide some insights about the reason for poor semen quality and fertility in crossbred bulls. Since the numbers of Sertoli cells are fixed around the time of puberty (Wang et al., 1989) and sperm production starts after puberty, we studied the proteomics of Sertoli and spermatogenic cells in peri-pubertal bulls. The aim was to study the differential proteomic profile of spermatogenic and Sertoli cells in crossbred (Holstein Friesian × Tharparkar) bulls in comparison to exotic (Holstein Friesian) and indigenous (Tharparkar) bulls.

## Materials and methods

### Experimental animals and their management

A total of 12 peri-pubertal bulls (age 10 months), four each from the three experimental groups, i.e., Holstein Friesian (HF- Exotic control group), Tharparkar (TP -Indigenous control group) and HF × Tharparkar crossbred (KF—Target group; 50–75% HF inheritance) maintained at Livestock Research Centre, NDRI, were utilized for the study. Up to 6 months of age, the calves were reared in groups and fed as per NRC recommendations. After 6 months of age, calves were transferred to semi loose housing system and fed with concentrate feed (@ 1.25 kg per day/bull), *ad libitum* green fodder and clean drinking water. All the experimental bulls were dewormed and vaccinated against common diseases as per the standard norms of the farm and were maintained under similar management conditions.

### Unilateral castration of bulls

Before castration, the health status, body weight and growth rate of the young bulls were examined and found within normal range. Immediately prior to castration, the bulls were sedated with xylazine hydrochloride (*Xylaxin, Indian Immunologicals*, India) at the dosage rate of 0.25 ml/50 kg body weight. The site of surgery was shaved and cleaned thoroughly with antiseptics. Then testis was locally infiltrated with 5–8 ml of 2% lignocaine (*Cadila Healthcare Ltd*., India) at the level of the spermatic cord. Incision was given at lower part of scrotum with the help of a surgical scalpel (B.P. blade no. 23). Right testis of each animal was exposed and the spermatic cord was ligated tightly using catgut [Size 3-0; *Stericat Gutstrings (P) Ltd*., India]. After ligation intact testicle was incised, removed and placed in individual sterile containers containing normal saline with penicillin streptomycin (*Sigma Aldrich*, USA). All the bulls were given due post-operative care as per standard veterinary protocol.

### Isolation of spermatogenic and sertoli cells from testicular tissues

Connective tissue around the testis was removed and the testis was then washed three times in 0.9% sterile normal saline containing penicillin streptomycin (*Sigma Aldrich*, USA). The tunica albuginea was removed from the testicular tissue and ~4–5 g of testicular parenchyma was transferred to a sterile 15 ml centrifuge tube (*Nunc*, Denmark). The tissue was minced into small pieces and suspended in Dulbecco's modified Eagle's medium (DMEM; *Sigma Aldrich*, USA).

The testicular cells were isolated from the tissue, through a consecutive enzymatic digestion procedure as described by Izadyar et al. (2002) with some modifications. Briefly, after washing with Dulbecco's phosphate buffered saline (DPBS; *Sigma Aldrich*, USA), the tissue was washed with DMEM. The seminiferous epithelial cells were dispersed using digestion with enzymes. For the 1st enzymatic digestion, minced seminiferous tissue were suspended in DMEM containing collagenase (*Sigma Aldrich*, USA)—1 mg/ml, hyaluronidase type II (*Sigma Aldrich*, USA)—1 mg/ml, deoxyribonuclease (DNase; *Sigma Aldrich*, USA)—5 μg/ml and trypsin (*Sigma Aldrich*, USA)—1 mg/ml, and incubated in shaker incubator at 37°C operated at 160 oscillation/minute for 45 min. After this, the dispersed tissue was collected and centrifuged at 1000 rpm for 2 min. The supernatant was discarded and the tissue pellet was washed once again with DMEM. For the second enzymatic digestion, the dispersed tissue was suspended in DMEM containing collagenase—1 mg/ml, hyaluronidase type II—1 mg/ml, and DNase—5 μg/ml and again incubated in shaker incubator at 37°C (160 oscillations/minute for 30 min). After this, 10% foetal bovine serum (*Hyclone*, Canada) was added immediately to prevent the further digestion of the tissue. The suspension was centrifuged at 1000 rpm for 2 min and the supernatant containing the spermatogonial cells, Sertoli cells, myeloid cells, and other cells of the seminiferous tubular tissue was collected in 15 ml centrifuge tube.

For enrichment of germ cell and Sertoli cells, the supernatant was filtered through a 60 μm and then a 41 μm nylon mesh filter (Merck Millipore, India). The filtered cell suspension was then transferred to 1.5 ml eppendorf tube (*Nunc*, Denmark) and centrifuged at 10,000 rpm for 1 min. The pellet was collected and dissolved in 0.5 ml DMEM for further fractionation of cells through Percoll discontinuous density gradient method. An iso-osmotic Percoll suspension was prepared containing 82.2% Percoll (*Sigma Aldrich*, USA) in DMEM (without additives), 0.6% bovine serum albumin (BSA) and DNase—45 μg/ml. A discontinuous density gradient was prepared by diluting the iso-osmotic Percoll suspension with DMEM, 0.7% BSA (*Sigma Aldrich*, USA) and 50 μg/ml DNase. The gradients were created by layering 1 ml each of 11, 19, 27, 35, and 43% Percoll with PBS in to a 15 ml centrifuge tube. The cell suspension was layered on top of the gradient in 500 μl DMEM, 0.7% BSA and 50 μg/ml DNase. This gradient was centrifuged at 1500 rpm for 30 min at 18° C. Then Sertoli cells were recovered from 19 to 27% and 35 to 43% gradient concentrations and spermatogenic cells were recovered from 27 to 35% gradient (Liu et al., 2011).

### Purity of isolated testicular cells

For estimation of the purity of the isolated cells, a Sertoli cell specific marker (Vimentin) was used. The FITC conjugated vimentin antibody (*Biorbyt*, United Kingdom) stock vial was reconstituted with 200 μl of dimethyl sulfoxide (DMSO; *Sigma Aldrich*, USA) solution and 10 μl of the stock solution was taken in 0.5 ml eppendorf tube and added 40 μl of PBS as working solution. Vimentin was used at 1:300 dilutions and the staining was done as per Ma et al. (2013). Briefly, 2 μl of cell suspension was taken in a 0.5 ml eppendorf tube and 8 μl of PBS was added to it and mixed thoroughly. Smear was made on clean grease free glass slide, air dried and fixed with methanol for 15 min, then washed with tap water for 2 min. After drying, 0.4% Triton X-100 was added on the smear for 45 min, washed with tap water, air dried and the smear was covered with 50 μl of vimentin antibody. The antibody treated slide was incubated in humidified box at 37°C for 4 h and washed in tap water. Then the slide was mounted using anti-fading mounting media having 1,4-diazabicyclo (2,2,2) octane (DABCO) and examined under fluorescent microscope (*Nikon, SMZ 100/SMZ 800*, Japan) at 562 nm wavelength. At least 200 cells were counted per smear to find out the proportion of Sertoli cells.

### Protein extraction from spermatogenic and sertoli cells

The protein fractions from the spermatogenic and Sertoli cells were extracted through sonication method. The washed spermatogenic and Sertoli cells were suspended in 500 μl PBS solution and protein fractions were extracted with a probe sonicator (*Bandelin Sonopuls*, USA) for 15 sec on ice repeated 5 times at intervals of 2 min. The sonicated suspensions were centrifuged at 10,000 rpm for 15 min at 4°C, and the supernatant containing protein fractions were used for further analysis. The proteins in the samples were precipitated by using 2D clean up kit (*GE Healthcare Ltd*., Sweden) and the protein concentrations were determined using Bradford assay (Bradford, 1976).

### Two dimensional difference gel electrophoresis (2D-DIGE)

Equal amount of proteins from the spermatogenic/Sertoli cells from all the bulls within each breed were pooled to form two pooled groups each. Total 50 μ g of protein from the each of the pooled samples were labeled with Cydye flours according to minimal labeling protocol provided by the manufacturer (*GE healthcare*, Sweden). Labeled protein samples of crossbred, HF/TP and one internal standard were pooled together and rehydrating stock solution (8 M urea, 2 M thiourea, 2% CHAPS, 0.002% bromophenol blue) was added to make up the final volume to 250 μ l. DTT and IPG buffer (pH 3–10) were added at a final concentration of 0.003 and 0.5%, respectively. After 15 h of rehydration, IPG strips (7 cm, pH 3–10) were subjected to iso-electric focusing in an Ettan IPGphor3 system (*GE Healthcare*, Sweden) for a total of 25,000 Volt-hours. Each electro focused strip was equilibrated, first with 10 ml of SDS equilibration buffer containing 10 mg/ml DTT for 15 min. This was followed by second equilibration with SDS equilibration buffer containing 25 mg/ml iodoacetamide for 15 min. The strips were then transferred onto 13% homogenous polyacrylamide gels cast on SE 600 Ruby gel apparatus (*Amersham Biosciences*, USA). The strips were overlaid with 0.5% agarose sealing solution (0.5% agarose, 0.002% bromophenol blue in Tris-glycine electrode buffer). Separation in SDS-PAGE was carried out with constant running current set at 15 mA per gel at 20°C for 30 min, followed by 30 mA per gel at 20°C until the bromophenol blue dye front ran off from the bottom of the gels.

### Image acquisition and analysis

Labeled proteins were visualized using a Typhoon TRIO variable mode imager (*Amersham Biosciences*, USA). Cy2 images were scanned with 488/520 nm, Cy 3 images were scanned with 532/580 nm and Cy5 images were scanned with 633/670 nm. All gels were scanned with a PMT setting of 750–800 V with 200 μm/pixel resolution. Images were cropped using Image-Quant™ v5.5 (*Amersham Biosciences*, USA) to remove areas extraneous to the gel image. Gel images were processed using DeCyder™ 2D version 7.0 (*Amersham Biosciences, USA*). The images were imported to differential in gel analysis (DIA) workspace to create different workspaces for each gel. The maximum number of spots for each co-detection procedure was set to 1000. The spots were co-detected and quantified automatically as 2-D DIGE image pairs, intrinsically linking the samples to its in-gel standard.

### Identification of differentially expressed proteins

Matched spots of interest were picked manually from the preparative gel. These spots were subjected to in gel digestion by trypsin. The spots were minced into small pieces, washed with ammonium bicarbonate (NH_4_HCO_3_) 50 mM and shrunk with acetonitrile. Reduction and alkylation were performed with β-mercaptoethanol 20 mM (56°C, 30 min), followed by 2-iodoacetamide 55 mM (20°C, 20 min, in the dark). Proteins were digested in a buffer containing 25 mM NH_4_HCO_3_, 5 mM calcium chloride, and ~20 ng/μl of trypsin (37°C, overnight). Peptides were extracted from gel in two steps by adding 25 mM NH_4_HCO_3_ and 5% formic acid; each step was followed by addition of acetonitrile to shrink the gel and maximize the peptide recovery.

Matrix-assisted laser desorption/ionization-Time of flight/Mass spectrometry (MALDI-TOF/MS) analysis was performed using an Ultraflex III TOF-TOF instrument (*Bruker Daltonics*, Germany), equipped with smart beam I laser (λ = 355 nm) and operating in reflectron positive ion mode. The instrumental conditions were: UIS1 = 25 kV; UIS2 = 21.65 kV; reflectron potential: 26.3 kV; delay time = 0 ns. The digested samples were dried, re-suspended in acetonitrile and desalted by Zip-Tip C18 pipette tips (*Merck Millipore*, Germany). Zip-tipped samples were spotted on the stainless steel sample plate with α-cyano-4-hydroxycinnamic acid matrix (5 mg in water/Acetonitrile/0.1% Trifluoro acetic acid). 1 μL of the peptide eluted solution was deposited on the stainless steel sample holder, and allowed to dry before introduction in to the mass spectrometer. External mass calibration was done using the Peptide Calibration Standard, based on the mono-isotopic values of [M+H]+ of Angiotensin II, Angiotensin I, Substance P, Bombesin, ACTH clip (1–17), ACTH clip (18–39), Somatostatin 28 at m/z. 1046.542, 1296.685, 1347.736, 1619.823, 2093.087, 2465.199 and 3147.471, respectively.

MALDI-TOF-TOF experiments were carried out using the LIFT device. The instrumental parameters were: UIS1 = 8 kV; UIS2 = 7.2 kV; ULIFT1 = 9 kV. The resulting data were processed using the Flex Analysis 2.4 software (*Bruker Daltonics*, Germany) and optimized for databank search. Searches were performed using the Mascot Server 2.3 search engine against the Swiss-Prot database (release, subset *Bos taurus*, entries) considering up to one missed tryptic cleavage, monoisotopic peptide mass tolerance of 500 ppm, and fragment ion mass tolerance of 0.2 Da. Carbamidomethyl modifications of cysteine and oxidation of methionine were considered as appropriate. Protein identifications were accepted if they had greater than 95% probability as represented by the mascot scores in the mascot result page.

## Results

The enzymatic digestion of 5 g testicular parenchyma tissue yielded ~300 million spermatogenic and Sertoli cells. The purity of the Sertoli and spermatogenic cells was estimated using antibodies against Vimentin through immunofluorescence. The purity of the Sertoli cells was 92% and of the spermatogenic cells was 94%.

### Differential proteomic profile of spermatogenic cells

**Figure 1:**
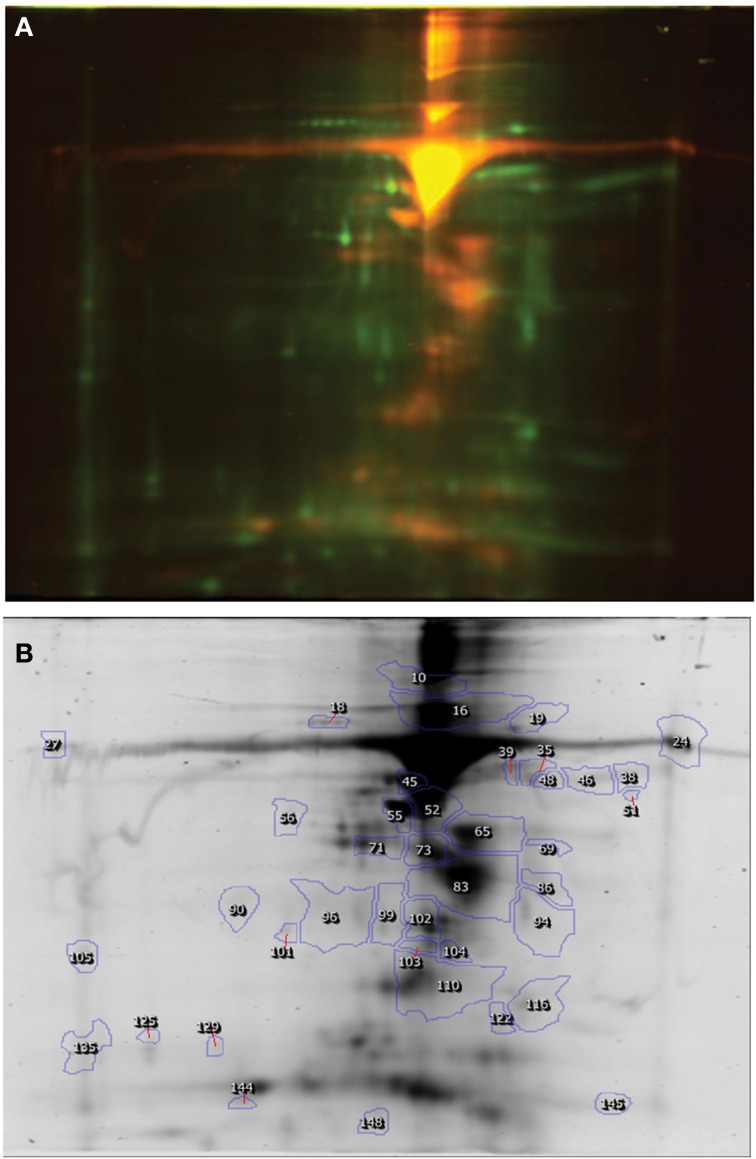
**DIGE image of Spermatogenic cell proteins (Holstein Friesian × Karan Fries). (A)** DIGE image, **(B)** Differentially expressed spots.

The proteomic analysis using Difference in Gel Electrophoresis (DIGE) and image scanning identified the presence of total 214 protein spots in a pooled sample of spermatogenic cells from HF and crossbred bulls (Figure 1). Among these, majority of the spots were differentially expressed between both the groups. For further study, spots having at least two fold differences in expression level between the groups were selected. Based on this, 14 proteins were found over expressed in the spermatogenic cells isolated from HF bulls, whereas 26 protein spots were under expressed in HF compared to crossbred bulls (Table 1). The differential expression of protein spots ranged from 3.1 to 36.9-folds between two breed groups.

**Table 1:** **Differentially expressed spots in the DIGE gel of spermatogenic cells (HF × KF) analyzed using the Decyder™ software**.

**Spot no**	**Fold difference**	**Average normalized volumes**	
		**HF**	**KF**
102	4.9	1.474	0.301
94	10.1	0.337	3.404
101	26.7	0.121	3.220
105	6.8	0.452	3.090
103	11.0	1.516	0.138
86	7.6	0.358	2.738
83	4.6	1.506	0.330
96	5.0	0.442	2.215
90	5.4	0.484	2.633
99	4.6	0.601	2.786
129	3.8	0.441	1.692
125	6.1	0.524	3.219
145	3.5	0.666	2.324
148	7.7	0.283	2.180
144	36.9	0.074	2.725
110	4.8	1.343	0.280
104	7.7	1.508	0.196
116	6.1	0.430	2.616
135	3.1	0.663	2.026
122	3.7	0.738	2.698
35	3.6	0.599	2.163
27	6.8	1.537	0.226
39	4.2	0.566	2.363
46	4.9	0.659	3.250
38	9.8	0.374	3.662
16	5.3	2.011	0.381
10	5.3	2.046	0.389
19	3.3	0.576	1.925
24	5.0	1.465	0.290
18	6.9	0.362	2.506
65	6.5	1.664	0.257
56	5.2	0.469	2.455
73	9.2	1.553	0.169
69	7.2	0.364	2.635
71	3.2	1.307	0.410
48	4.2	0.717	2.991
45	6.1	3.287	0.538
52	4.5	2.628	0.589
55	5.2	1.772	0.341
51	11.1	0.235	2.618

**Figure 2:**
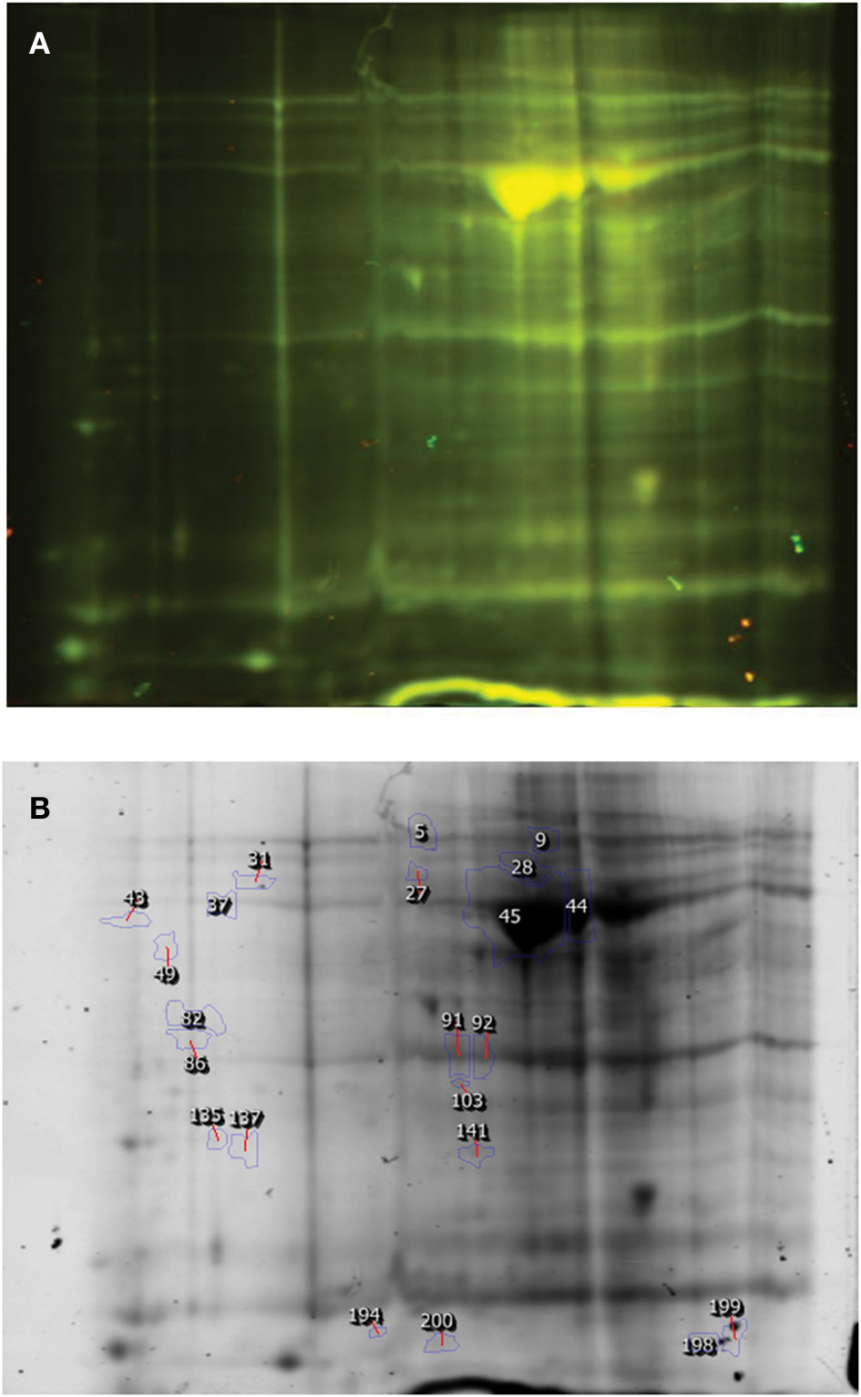
**DIGE image of Spermatogenic cell proteins (Karan Fries × Tbarparkar). (A)** DIGE image, **(B)** Differentially expressed spots.

The DIGE image analysis of proteins from spermatogenic cells of TP and crossbred bulls are presented in Figure 2 and Table 2. It was found that out of total 219 spots detected, 7 protein spots were over expressed and 15 protein spots were under expressed in the spermatogenic cells of TP bulls compared to that of crossbred bulls in 2-fold or above range. The expression level varied from 2.0 to 23.8 between the groups.

**Table 2:** **Differentially expressed spots in the DIGE gel of spermatogenic cells (KF × TP) analyzed using the Decyder™ software**.

**Spot no**	**Fold difference**	**Average normalized volumes**	
		**KF**	**TP**
103	2.1	0.552	1.145
135	2.3	1.575	0.696
92	2.3	0.495	1.114
86	2.0	1.555	0.766
91	2.1	0.530	1.089
137	2.5	2.213	0.876
200	2.0	0.535	1.072
198	7.5	4.439	0.592
194	9.2	4.123	0.447
141	2.1	0.593	1.245
199	23.8	7.621	0.320
27	3.1	2.365	0.772
45	2.2	1.715	0.788
28	2.6	2.043	0.794
5	2.1	0.508	1.052
9	2.8	0.387	1.099
44	2.0	1.575	0.779
49	2.2	2.281	1.038
82	2.8	2.593	0.914
43	2.2	1.913	0.882
31	10.3	6.497	0.632
37	2.3	2.236	0.987

### Differential proteomic profile of sertoli cells

**Figure 3:**
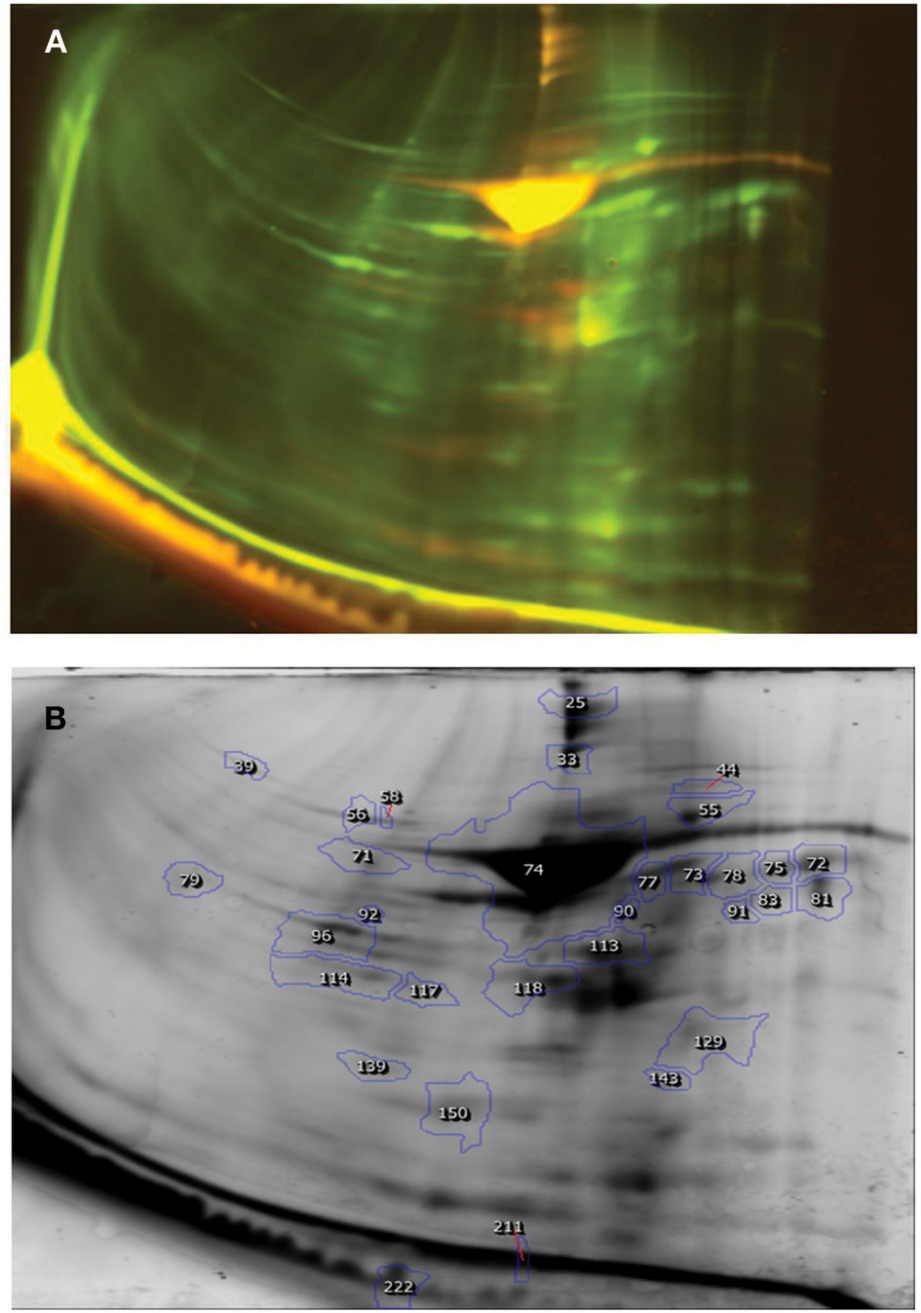
**DIGE image of Sertoli cell proteins (Holstein Friesian × Karan Fries). (A)** DIGE image, **(B)** Differentially expressed spots.

A total of 176 protein spots were detected in the DIGE image of Sertoli cell proteins from HF and crossbred bulls (Figure 3, Table 3). Among these, it was observed that 8 proteins spots were over expressed and 23 spots were under expressed in the DIGE profile of Sertoli cells of HF bulls compared to their crossbred counter parts in a threefold range or above. The expression level varied from 3.1 to 9.4 between two groups. Since the quantity of isolated Sertoli cells was very less, we could not compare the proteomic profile of Sertoli cells between crossbred and TP.

**Table 3:** **Differentially expressed spots in the DIGE gel of Sertoli cells (HF × KF) analyzed using the Decyder™ software**.

**Spot no**	**Fold difference**	**Average normalized volumes**	
		**HF**	**KF**
96	3.1	0.448	1.382
113	3.2	1.759	0.555
114	4.5	0.340	1.519
92	4.7	0.353	1.673
83	3.6	0.438	1.567
90	6.2	0.356	2.212
91	4.4	0.351	1.558
118	7.5	1.861	0.248
150	3.5	1.837	0.518
211	3.4	2.757	0.821
222	4.1	2.075	0.503
143	3.5	0.443	1.562
117	4.7	0.310	1.467
129	3.7	0.440	1.609
139	5.5	0.266	1.457
81	5.0	0.306	1.539
74	4.3	2.667	0.621
55	4.1	0.398	1.615
56	6.4	0.206	1.313
44	3.4	0.463	1.552
25	6.1	2.119	0.345
33	5.9	2.270	0.387
39	4.5	0.334	1.499
58	7.9	0.168	1.320
73	4.9	0.358	1.740
79	4.9	0.370	1.831
77	6.9	0.277	1.903
75	6.1	0.265	1.605
71	9.4	0.154	1.456
72	6.3	0.268	1.685
78	5.2	0.300	1.558

### Selection and identification of some differentially expressed proteins

After DIGE image analysis, from the panel of differentially expressed proteins, based on the magnitude of differential expression and visibility after silver staining, 12 protein spots were selected for further identification through MALDI-TOF/MS. DIGE has very high sensitivity for proteins and large number of protein spots were visible, but upon silver staining limited number of spots could only be analyzed. Out of these 12 proteins, 4 proteins each were belonged to the three experimental samples, i.e., from spermatogenic cells of HF × crossbred and TP × crossbred and Sertoli cells of HF × crossbred. The details of the identified proteins along with the Mascot score are presented in Table 4. For each protein match, Mascot calculates an overall Protein Score. This number reflects the combined scores of all observed mass spectra that can be matched to amino acid sequences within that protein. A higher score indicates a more confident match. The spots 1 to 7 were identified as albumin, and the spot 8 was identified as Phosphatidyl ethanolamine Binding Protein (PEBP), which was over expressed in the spermatogenic cells of crossbred bulls compared to TP with a 2-fold increase in expression. Among the four selected spots of Sertoli cells, spots 10 and 11 were identified as albumin, whereas spots 9 and 12 were identified as Gamma Actin (γ-Actin) and RINGO/Speedy protein-A, respectively, (Table 4). γ-Actin was found to be over expressed in the Sertoli cells of HF bulls, whereas Speedy Protein A was found to be over expressed in crossbred bulls.

**Table 4:** **Details of differentially expressed proteins identified through MALDI-TOF/MS**.

**No**	**Sample**	Breeds^*^	**Protein**	**Accession (NCBI)**	**Mw (Da)**	**MASCOT score**	**Peptide match**	**Over expressed in**
1	Spermatogenic cells	HF × KF	Albumin	gi|30794280	71,274	143	17	HF
2		HF × KF	Albumin	gi|30794280	71,274	137	12	HF
3		HF × KF	Albumin	gi|76445989	55,487	115	13	HF
4		HF × KF	Albumin	gi|30794280	71,274	83	15	HF
5		KF × TP	Albumin	gi|30794280	71,274	293	31	KF
6		KF × TP	Albumin	gi|30794280	71,274	107	17	KF
7		KF × TP	Albumin	gi|30794280	71,274	182	24	KF
8		KF × TP	Phosphatidyl ethanolamine binding protein	gi|4389366	20,828	76	8	KF
9	Sertoli cells	HF × KF	**γ**-Actin	gi|809561	41,335	130	17	HF
10		HF × KF	Albumin	gi|30794280	71,274	148	21	HF
11		HF × KF	Albumin	gi|30794280	71,274	131	18	HF
12		HF × KF	Speedy Protein A	gi| 217330630	36,547	66	17	KF

* HF, Holstein Friesian;

KF, Crossbred; TP, Tharparkar.

## Discussion

The reason for higher incidence of sub-fertility and poor semen quality in crossbred bulls compared to purebred bulls is not yet fully understood at present despite the fact crossbreeding is used very commonly to improve the milk productivity. Proteomic differences in the spermatozoa (Peddinti et al., 2008; Park et al., 2012; Soggiu et al., 2013) and seminal plasma (Killian et al., 1993; Moura, 2005) between high and low fertile bulls have been investigated and putative markers for fertility have been proposed. However, the possible differences at the sperm precursor cells and supporting/nourishing cells in between crossbred and purebred bulls have not been studied. In the present paper, we report the proteomic differences in Sertoli and spermatogenic cells isolated from crossbred and purebred bulls. To the best of our knowledge, this is the first study analyzing the expression level differences in the proteomic profile of spermatogenic and Sertoli cells between crossbred and purebred bulls.

Expression levels of several proteins in Sertoli and spermatogenic cells differed between purebred and crossbred bulls. Compared to HF bulls, 26 proteins were over expressed and 14 proteins were under expressed in spermatogenic cells of crossbred bulls. Similarly, 7 protein spots were over expressed and 15 protein spots were under expressed in the spermatogenic cells of TP bulls compared to that of crossbred bulls. It has been reported that the differentiation of spermatogenic cells involves profound changes in the proteome, and the ultra-structure of the nucleus, flagellum, mitochondria, and Golgi (Eddy and O'Brien, 1998) and 35 proteins were differentially expressed among diploid spermatogonia, tetraploid spermatocytes and the haploid spermatids (Rolland et al., 2009). Among the several differentially expressed proteins in the spermatogenic cells, we identified that PEBP was over expressed in crossbred bulls compared to TP bulls. PEBP, a 187 amino acid containing protein, belongs to a family of phospholipid-binding proteins, has been described previously in male reproductive tract, where it has been implicated in the biogenesis and maintenance of antigen segregation of membranes (Moore et al., 1996). They are an evolutionarily conserved family of proteins implicated in mitogen-activated protein (MAP) kinase pathway regulation. PEBP-2 is the testis-specific protein found within late meiotic and haploid germ cells in a stage-specific pattern with specific roles in spermatogenesis and post-testicular sperm maturation. In addition to signal transduction, they are also involved in the development of functional cell membrane domains or as an inhibitor of serine proteases involved in spermiogenesis or post-testicular sperm maturation (Hickox et al., 2002). In adult rat testis, PEBP was localized in elongating spermatids, residual bodies, and interstitial Leydig cells. In pre-pubertal animals, PEBP was expressed in both testes and epididymis and was localized to Leydig cells from day 1 of post-natal life and was not detected in any other cell type until the differentiation of elongated spermatids (Moore et al., 1996). It was also reported that PEBP may be involved in the organization of sperm membranes during spermiogenesis (Frayne et al., 1998). Recently, from our laboratory, it was found out that the number of spermatids was higher in good bulls compared to poor bulls (Rajak, 2012). Less percentage of spermatids in poor bulls suggested the existence of a partial maturation of germ cells (Foresta and Varotto, 1992). Since the PEBP proteins are mostly confined to the spermatids, the number of spermatids may be significantly higher in crossbred bulls leading to increased expression of these proteins in spermatogenic cells of crossbred bulls.

Sertoli cells are the key element in the tight regulation of the highly organized progression of the spermatogenesis. Sertoli cells produce various factors that affect the balance between differentiation, self renewal and maturation of germ cells. Germ cell and Sertoli cells are interrelated in their mutual control and physiological functions (Russell et al., 1990; Dadoune, 1993). We observed that testicular γ-Actin protein is over expressed in the Sertoli cells of HF bulls compared to crossbred bulls. It is already reported that γ-Actin is present within apical processes of Sertoli cells processes encapsulating the heads of late step spermatids (Oko et al., 1991) and plays a key role in maturation and differentiation of germ cells (Cavicchia et al., 2011). It is also reported that the expression of γ-Actin in Sertoli cells is related to semen quality parameters. Giesecke et al. (2010) also reported that genes encoding for a, b, and c actins (ACTN, ACTB, ACTG), are potential candidates for association analysis of fertility. From the available literature, it is evident that γ-Actin is responsible for the structural integrity and functional ability of the Sertoli cell and germ cell junctions, and the present study showed that these proteins are under expressed in the Sertoli cells of crossbred bulls. It is possible that the reduced level of γ-Actin may affect the Sertoli cell- germ cell interactions leading to defective nourishment of spermatogenic cells and subsequently low spermatogenic efficiency in crossbred bulls.

RINGO/Speedy-A is a mammalian protein that activates cyclin-dependent kinases (CDKs) and play important roles in cell cycle progression. They are extensively present in the testicular tissue implying their function during meiosis. Over expression of a Speedy-A fused to GFP impairs cell cycle progression. In particular, a high percentage of the cells expressing GFP-Speedy-A at late stages of mitosis round up and stop cycling (Dinarina et al., 2009). Overexpression of a stabilized Speedy-A form results in the accumulation of high levels of Speedy-A at late stages of mitosis, which interfere with cytokinesis and chromosome de-condensation (Dinarina et al., 2009). Another study showed that a human homolog of Speedy protein is essential for S-phase entry in a Cdk2- dependent manner. Over-expressions of human Speedy protein accelerate S-phase entry and cell proliferation and its inhibition by RNAi caused a cell cycle arrest at G1/S in cultured cells (Porter et al., 2002).The available literature showed that over expression of Speedy-A causes alterations in cell cycle. In present study, it was noticed that the Sertoli cells of crossbred bulls had over expression of Speedy-A protein. This over expression might be causing cell cycle arrests in the some germ cells of crossbred bulls, compared to exotic or indigenous bulls.

Several reports indicate that differential expression of specific proteins could alter sperm functions, jeopardizing its fertilizing abilities, thus altering the fertility (Peddinti et al., 2008; D'Amours et al., 2010; Gaviraghi et al., 2010). According to Feugang et al. (2009), 30 proteins were increased, and 27 were decreased in low fertile bulls compared to high fertile bulls within a twofold range. Wang et al. (2010) also identified 18 sperm proteins that varied significantly, between high and low fertile Angus bulls. Taken together the published reports on sperm proteomics and the results of the present study, it is evident that certain proteomic level differences exist in sperm precursor cells and nourishing cells between breeds with different fertility potential.

## Conclusion

Our initial findings suggest the existence of differences in the proteomic profile of Sertoli and spermatogenic cells in crossbred bulls in comparison to their exotic and indigenous parentage. Since a considerable proportion of crossbred bulls are reported to be sub-fertile compared to their exotic and indigenous parent lines, the findings of the present study are of value in improving our understanding on the underlying reasons for sub-fertility in this species.

### Conflict of interest statement

The authors declare that the research was conducted in the absence of any commercial or financial relationships that could be construed as a potential conflict of interest.
